# Supplementary material for: Optimal evaluation of energy yield and driving force in microbial metabolic pathway variants
Source: PLoS Comput Biol. 2023 Jul 6;19(7):e1011264. doi: 10.1371/journal.pcbi.1011264 (PMC10353829; doi:10.1371/journal.pcbi.1011264)
Supplement: S1 Text — (PDF) [file pcbi.1011264.s001.pdf]

SUPPLEMENTARY INFORMATION:
Optimal evaluation of energy yield and driving force in microbial metabolic pathway variants

Ahmed Taha[1], David R Penas[2], Mauricio Patón[1], Julio R Banga[2], and Jorge Rodríguez[1*]

[1] Department of Chemical Engineering, Research and Innovation Center on CO_2 and H_2 (RICH) Khalifa University. PO Box 127788, Abu Dhabi, United Arab Emirates

[2] Computational Biology Lab, MBG-CSIC (Spanish National Research Council), 36143 Pontevedra, Galicia, Spain

[*]Correspondence: *jorge.rodriguez@ku.ac.ae*; Tel.: +971 2 312 4250

1. Acid-Base Equilibria

The pH-based speciation of acidic species occurs at a timescale that is several orders of magnitude faster than metabolic reactions, and thus it is typical to assume those species to be at equilibrium. This implies that the protonated form of a species and all its successive deprotonated forms are at the same chemical potential, and thus the choice of microspecies does not affect the rest of the framework constraints. The underlying assumption in such a situation is that the bounds described in section 2.5 are applied to the total concentration of each species rather than individual microspecies.

However, it may be desired in some models to select one species as the true substrate of the metabolic reactions and apply the concentration bounds to that form. Furthermore, different reactions in the same pathway may use two different forms as the true substrate, meaning the two species concentrations have to be included as decision variables, but are not independent of one another. An example of this is the dicarboxylate-4-hydroxybutyrate cycle in autotrophic CO_2 fixing archaea, where both CO_2 and HCO_3^- are true substrates for different reactions in the pathway [1]. In such a case, it becomes necessary to introduce an equality constrain based on the definition of acid dissociation constant:

$$K_a = \frac{C_{H^+} * C_{A^-}}{C_{HA}} \tag{S1}$$

where HA is the protonated form of a species, A^- is its deprotonated form, and K_a is the acid dissociation constant associated with the deprotonation. Fortunately, by applying the logarithm to equation (17), we can obtain a linear equality constrain:

$$\ln C_{A^-} - \ln C_{HA} = \ln 10 * (pH - pK_a) \tag{S2}$$

2. Case study pathway information

The following reaction stoichiometries, possibility of proton translocation and permissible electron carriers have been assembled using previous work [2] and biochemical databases [3,4]. Note that the molar ratio of the electron carrier regeneration reactions vary depending on the variant being evaluated.

Table A: Reaction and biochemical information for propionate oxidation pathways. The numbering of the pathways is as presented in the figure in the main text.

Reaction	Molar Ratio						SLP	Prot trans allowed?	Electron Carriers
	P1	P2	P3	P4	P5	P6			
Propanoic Transport (In)	1	1	1	1	1	1	0	Yes	
Propionate -> Propionyl-CoA	1	1	0	0	1	0	-1	No	
Propionate + Acetyl-CoA -> Acetate + Propionyl-CoA	0	0	1	0	0	1	0	No	
Propionyl-CoA -> methylMalonyl-CoA	1	0	1	0	0	0	-1	Yes	
Propionyl-CoA + Oxaloacetate -> methylMalonyl-CoA + Pyruvate	0	1	0	0	0	0	0	No	
mMalon-CoA -> Succinyl-CoA	1	1	1	0	0	0	0	No	
Succinyl-CoA -> Succinate	1	1	1	0	0	0	1	No	
Succinate -> Fumarate	1	1	1	0	0	0	0	Yes	FAD, Quinone
Fumarate -> Malate	1	1	1	0	0	0	0	No	
Malate -> Oxaloacetate	1	1	1	0	0	0	0	No	NAD, FAD
Oxaloacetate -> Pyruvate	1	0	1	0	0	0	1	Yes	
Pyruvate -> Acetyl-CoA	1	1	1	1	0	0	0	No	Fd
Propionate + Lactoyl-CoA -> Lactate + Propionyl-CoA	0	0	0	1	0	0	0	No	
Propionyl-CoA -> Acryloyl-CoA	0	0	0	1	1	1	0	Yes	FAD, Quinone
Acryloyl-CoA -> Lactoyl-CoA	0	0	0	1	0	0	0	No	
Lactate -> Pyruvate	0	0	0	1	0	0	0	No	NAD
Acryloyl-CoA -> 3-hydroxyPropionyl-CoA	0	0	0	0	1	1	0	No	
3-hydroxyPropionyl-CoA -> 3-hydroxyPropionate	0	0	0	0	1	1	1	No	
3-hydroxypropionate -> Malonate semialdehyde	0	0	0	0	1	1	0	No	NAD
Malonate semialdehyde -> Acetyl-CoA	0	0	0	0	1	1	0	No	NADP
Acetyl-CoA -> Acetate	1	1	0	1	1	0	1	No	
Acetate Transport (Out)	1	1	1	1	1	1	0	Yes	
$FADH_2 + NAD^+$ -> FAD^{2+} + NADH							0	Yes	
$UQ_{red} + NAD^+$ -> UQ_{ox} + NADH							0	Yes	
Fd_{red} -> $Fd_{ox} + H_2$							0	Yes	
NADH -> $NAD^+ + H_2$							0	Yes	
NADPH -> $NADP^+ + H_2$							0	Yes	

Table B: Reaction and biochemical information for the reverse TCA cycle pathway. Note that the molar ratios for the electron carrier reactions will be negative, indicating that they will proceed in the opposite direction to the one listed in the table

Reaction	Molar Ratio	SLP	Prot trans allowed?	Electron Carriers
CO2 Transport	2	0	Yes	
Succinyl CoA -> 2-ketoglutarate	1	0	No	NAD, NADP, Fd
2-ketoglutarate -> Isocitrate	1	0	Yes	NAD, NADP
Isocitrate -> Aconitate	1	0	No	
Aconitate -> Citrate	1	0	No	
Citrate -> Oxaloacetate + Acetyl CoA	1	-1	No	
Oxaloacetate -> Malate	1	0	Yes	NAD, NADP, Quinone
Malate -> Fumarate	1	0	No	
Fumarate -> Succinate	1	0	Yes	NAD, FAD, Quinone
Succinate -> Succinyl CoA	1	-1	No	
$FADH_2 + NAD^+$ -> FAD^{2+} + NADH		0	Yes	
$UQ_{red} + NAD^+$ -> UQ_{ox} + NADH		0	Yes	
Fd_{red} -> $Fd_{ox} + H_2$		0	Yes	
NADH -> $NAD^+ + H_2$		0	Yes	
NADPH -> $NADP^+ + H_2$		0	Yes	

3. Thermodynamic information

Table C: Standard enthalpies and Gibbs energy changes of formation for species used in this work

Species Name	ΔG^0_f (kJ/mol)	ΔH^0_f (kJ/mol)	Source
Propionate	-361.08	-510.4	[2]
Succinyl CoA	-461.89	-619.32	[5]
Coenzyme A	0	0	[5]
Acetyl CoA	-140.67	-227.77	
2-ketoglutarate	-793.41	-1044.1	[5]
iso-citrate	-1156	N/A	[5]
cis-Aconitate	-917.13	N/A	[5]
Citrate	-1162.7	-1515.1	[5]
Oxaloacetate	-793.82	-959.9	[5]
Malate	-843.22	-1079.8	[6]
Fumarate	-602.27	-777.91	[6]
Succinate	-690.91	-909.29	[7]
Pyruvate	-472.27	-596.22	[6]
Malonate semialdehyde	-477.6	-609.39	[5]
3-hydroxypropionate	-518.4	-676.04	[5]
Hydroxypropionyl-CoA	-285	-400.64	[5]
Acryloyl CoA	-48.5	-122.98	[8]
Propionyl CoA	-131.31	-248.41	[5]
Methylmalonyl CoA	-454.65	-624.6	[5]
Lactate	-517.81	-687	[2]
Lactoyl CoA	-287.8	-405.92	[8]
Hydrogen	17.55	-4.16	[8]
Acetate	-369.41	-486	[2]
Ubiquinone (ox)	0	N/A	[5]
Ubiquinone (red)	-64.43	N/A	[5]
Ferredoxin (ox)	0	N/A	[7]
Ferredoxin (red)	79.1177	N/A	[7]
FAD	0	N/A	[7]
FADH2	-37.4	N/A	[7]
NAD+	0	N/A	[2]
NADH	21.83	N/A	[2]
NADP+	0	NA	[2]
NADPH	21.83	NA	[2]
Carbon Dioxide	-386	-413.8	[2]
Proton	0	0	
Water	-237.18	-285.8	[5]

4. Multiplicity of solutions to the optimization problem

As discussed in section 3.1.1 of the main text, the concentration profiles and configuration of proton translocations obtained by the solvers for an optimized pathway variant is not unique. Thus, there are several profiles that lead to the same optimum energy recovery and driving force distribution. In the figures below, we present multiple such profiles for the cyclical hydroxypropionyl pathway.

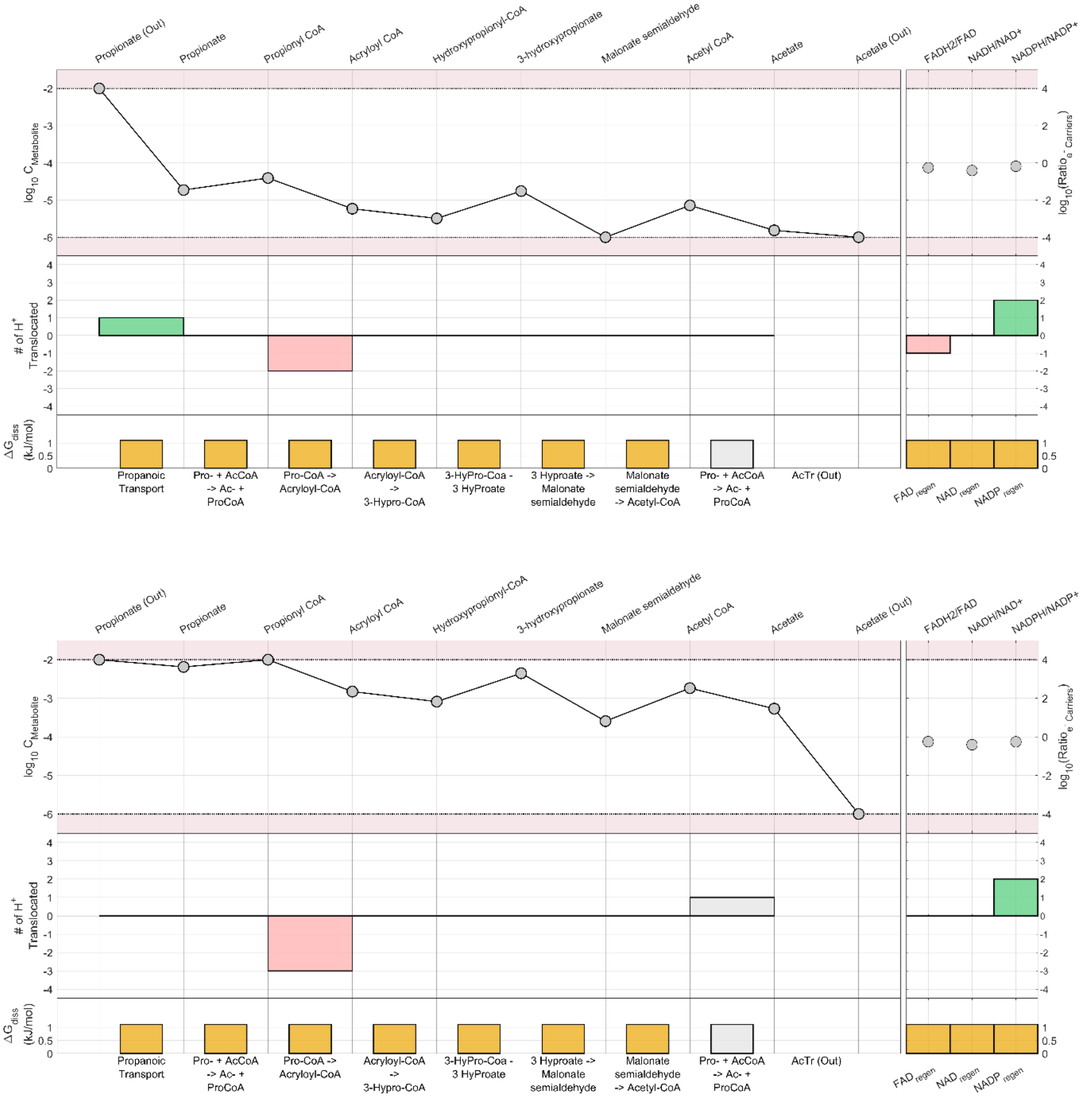

Fig **A**. Two additional possible concentration profiles leading to optimum yield and driving forces in the hyroxypropionyl CoA pathway for the conditions simulated in the main text Table 3

References

1. Bar-Even A, Noor E, Milo R. A survey of carbon fixation pathways through a quantitative lens. J Exp Bot. 2012;63: 2325–2342. doi:10.1093/jxb/err417

2. Patón M, Hernández HH, Rodríguez J. Comprehensive Bioenergetic Evaluation of Microbial Pathway Variants in Syntrophic Propionate Oxidation. mSystems. 2020;5: e00814-20. doi:10.1128/mSystems.00814-20

3. Caspi R, Altman T, Billington R, Dreher K, Foerster H, Fulcher CA, et al. The MetaCyc database of metabolic pathways and enzymes and the BioCyc collection of Pathway/Genome Databases. Nucleic Acids Research. 2014;42: D459–D471. doi:10.1093/nar/gkt1103

4. Kanehisa M, Goto S. KEGG: kyoto encyclopedia of genes and genomes. Nucleic Acids Res. 2000;28: 27–30. doi:10.1093/nar/28.1.27

5. Alberty RA. Biochemical Thermodynamics: Applications of Mathematica. John Wiley & Sons; 2006.

6. Heijnen J. Bioenergetics of Microbial Growth. In: Flickinger M, Drew S, editors. Encyclopedia of Bioprocess technology: fermentation, biocatalysis and bioseparation. Chichester UK: John Wiley & Sons; 1999. pp. 267–291.

7. Thauer RK, Jungermann K, Decker K. Energy conservation in chemotrophic anaerobic bacteria. 1977;41: 81.

8. Hanselmann KW. Microbial energetics applied to waste repositories. Experientia. 1991;47: 645–687. doi:10.1007/BF01958816
