# Supplementary material for: Optimal evaluation of energy yield and driving force in microbial metabolic pathway variants
Source: PLoS Comput Biol. 2023 Jul 6;19(7):e1011264. doi: 10.1371/journal.pcbi.1011264 (PMC10353829; doi:10.1371/journal.pcbi.1011264)
Supplement: Attachment — Submitted filename: report_PCOMPBIOL-D-23-00252.pdf [file pcbi.1011264.s002.pdf]

Review of manuscript PCOMPBIOL-D-23-00252

General comments:

This submission provides a new methodology to investigate pathway selection mechanisms in microbial populations, based on energetic considerations. In my opinion, the manuscript is well written and the technical aspects are described in such a way that they are accessible to the non-specialist.

My main concern is about conclusions relative to kinetic aspects and the classification of reactions as rate-limiting reactions. As far as I understood, enzyme regulation is not taken into account in the flux-force efficacy or the minimum driving force. For example, in the case of glycolysis/gluconeogenesis, some thermodynamically irreversible reactions of glycolysis are catalysed by a different enzyme to proceed in the gluconeogenesis direction, and the metabolites concentrations determine which enzyme is active (resp. inactive). So, my question is: does the developed methodology implicitly account for enzyme regulation? If yes, it should be explained in more details. If not, how would the conclusions of the present manuscript be affected if enzymatic regulations were taken into account?

Some minor comments and questions**:**

l. 195: I could not find the discussion about activity in section 1. I think the authors mean section 2.3 (in the paragraph about the other assumptions of the approach).

l. 221: the proton motive force is usually not defined as a ratio, but as a force depending on a membrane potential and on a pH difference. The definition given in the manuscript is thus a bit misleading. Also, we can see in Tables 3 and 4 that the pH difference is taken as 0 in some simulations (pH_in = pH_out), but is it always the case? If not, is r_H+/ATP changed accordingly? Then, is the value 9/3 accounting for slippage or does it assume ideal coupling between ATP synthesis and proton translocation?

l. 276 (Fig. 5): some reaction arrows and couples of cofactors / cosubstrates are shown in the wrong directions. For example, for succinate → succinyl-CoA, ATP is indeed consumed by the reaction but the arrow does currently not point to the same direction as the main reaction arrow. The same issue arises for succinyl-CoA → 2-ketoglutarate and 2-ketoglutarate → isocitrate.

l. 394: the authors probably mean "boosts" and not "boasts"

l. 417: a word is probably missing between "approach" and "in"

l. 458 (Table 3): I was puzzled by the fact that the performances of propionate oxidation pathways via lactate or via hydroxypropionyl CoA cyclical are exactly the same. Can the authors provide a mechanistic explanation about this result?

l. 486 (Fig. 3): if I understand correctly, Fig. 3 and 7 were obtained by changing the value of epsilon in equation (19). Would it be relevant to indicate the values of epsilon in the Figure?

In the last part of the manuscript (sections 3 and 4), the authors may want to switch to British English (used in the first part of the manuscript) instead of American English.
