# Supplementary material for: Optimal evaluation of energy yield and driving force in microbial metabolic pathway variants
Source: PLoS Comput Biol. 2023 Jul 6;19(7):e1011264. doi: 10.1371/journal.pcbi.1011264 (PMC10353829; doi:10.1371/journal.pcbi.1011264)
Supplement: Attachment — Submitted filename: report_PCOMPBIOL-D-23-00252-1.pdf [file pcbi.1011264.s004.pdf]

Review of manuscript PCOMPBIOL-D-23-00252-1

The authors have addressed my questions and the manuscript is in my opinion well improved, but I think that further clarification is needed for the following points.

First of all, I think that including a discussion about regulation in the conclusion could be of general interest and could make the applicability of the method to other pathways more credible, as enzyme regulation of metabolism is ubiquitous. While it is obvious that an enzyme cannot speed up the rate of a reaction that has no driving force, my question was more about the identification of rate-limiting reaction based on pure thermodynamic considerations (*i.e.* neglecting enzyme regulation that can also be at the source of rate-limiting reactions). From the reply of the authors, I understand that enzyme regulation is not relevant for the pathways considered and the only rate-limiting effect is thus thermodynamic, but I believe that it should be stated explicitly in the paper – for example, that could be clarified on line 611, where the authors mention that "since the approach is based entirely on thermodynamic principles, the pathway analysis results were obtained with minimum need for assumptions and/or parameter estimations." Then what about pathways where regulation becomes relevant? Since the method is said to be "maintaining a high degree of flexibility to accommodate a large range of additional constraints", I think at least a brief comment could be written about possible extensions accounting for regulation.

Secondly, the authors say in the response letter that "The value of $r_{H+/ATP}$ used for all results shown in the paper is 10/3", but in the manuscript, this value does not appear and 9/3 is mentioned instead (l. 237).
