# Supplementary material for: Optimal evaluation of energy yield and driving force in microbial metabolic pathway variants
Source: PLoS Comput Biol. 2023 Jul 6;19(7):e1011264. doi: 10.1371/journal.pcbi.1011264 (PMC10353829; doi:10.1371/journal.pcbi.1011264)
Supplement: Attachment — Submitted filename: Response to reviewers.docx [file pcbi.1011264.s003.docx]

The authors would like to thank the reviewers and editor for the time and effort in reviewing the manuscript. We have found all the comments useful and valuable and we believe they have contributed to increasing the quality of the revised manuscript. Below we provide detailed responses and indications of the changes as suggested

***Editor:***

*Two experts in thermodynamics have reviewed your submission. Based on their feedback, major revisions are required. You will see, however, that the requested revisions are not burdensome but I do ask that you take them seriously as it is important to respond to these in a diligent manner. One comment is in regard to characterizing the minimum driving force using shadow prices. While it would be fine to include such an analysis, I believe that it is only necessary to mention that this is possible and give references; using thermodynamics to characterize driving forces is always preferable to borrowing economic principles.*

We thank the editor for the valuable feedback on the manuscript. The authors have made the best effort to address all comments and concerns diligently. In particular, we highlight that shadow prices are only applicable to continuous optimisation problems and not to MILP. Reference to alternative methods of computing bottlenecks is made in the conclusions section (line 612-614).

***Reviewer 1:***

*This submission provides a new methodology to investigate pathway selection mechanisms in microbial populations, based on energetic considerations. In my opinion, the manuscript is well written and the technical aspects are described in such a way that they are accessible to the non-specialist.*

*My main concern is about conclusions relative to kinetic aspects and the classification of reactions as rate-limiting reactions. As far as I understood, enzyme regulation is not taken into account in the flux-force efficacy or the minimum driving force. For example, in the case of glycolysis/gluconeogenesis, some thermodynamically irreversible reactions of glycolysis are catalysed by a different enzyme to proceed in the gluconeogenesis direction, and the metabolites concentrations determine which enzyme is active (resp. inactive). So, my question is: does the developed methodology implicitly account for enzyme regulation? If yes, it should be explained in more details. If not, how would the conclusions of the present manuscript be affected if enzymatic regulations were taken into account?*

The biochemical aspects considered are limited in this example to the reaction steps with no specific regulation. Regulation could arguably be seen as an additional biochemical tool of the cell to control, reactions towards a form of optimality, however arguably in systems under no energy limitation. Irrespective of regulation there is a need for a driving force and in energy limited pathways this becomes the critical aspect. Regulation could slow down but hardly speed up a reaction with no driving force.

Differences between enzymes and possibly regulation could be implemented in the model by forcing more stringent driving force constraints for enzymes with known slower kinetic parameters.

*Some minor comments and questions:*

*l. 195: I could not find the discussion about activity in section 1. I think the authors mean section 2.3 (in the paragraph about the other assumptions of the approach).*

This was indeed an overlook. The reference about activity assumptions has been corrected in the text to section (2.3)

*l. 221: the proton motive force is usually not defined as a ratio, but as a force depending on a membrane potential and on a pH difference. The definition given in the manuscript is thus a bit misleading. Also, we can see in Tables 3 and 4 that the pH difference is taken as 0 in some simulations (pH_in = pH_out), but is it always the case? If not, is r_H+/ATP changed accordingly? Then, is the value 9/3 accounting for slippage or does it assume ideal coupling between ATP synthesis and proton translocation?*

While we acknowledge the common definition of pmf as a sum of chemical potential and electric potential gradients, we opted for the definition that relates it to the main energy token (ATP) as it better serves the aims of this work, allowing us to express proton translocations and SLP in a single overall energy recovery unit. The value for r_H+/ATP_ is a biochemical parameter associated to ATP_ase_ complex and can be set to any reasonable value near 10/3 in the model. In addition, cells are known to keep the pmf relatively constant due to the above by adjusting the electrical potential when ΔpH is there. The value of r_H+/ATP_ used for all results shown in the paper is 10/3. Any other reporter integer number per ATP_ase_ turn (making 3 ATP) could be used like we did in (Paton et al. 2020) e.g. from 8/3 to 15/3 have been reported.

*l. 276 (Fig. 5): some reaction arrows and couples of cofactors / cosubstrates are shown in the wrong directions. For example, for succinate → succinyl-CoA, ATP is indeed consumed by the reaction but the arrow does currently not point to the same direction as the main reaction arrow. The same issue arises for succinyl-CoA → 2-ketoglutarate and 2- ketoglutarate → isocitrate.*

We thank the reviewer for spotting this error in the figure; it has been corrected. This error was only in the figures and not present in the model simulations.

*l. 394: the authors probably mean "boosts" and not "boasts"*

The intended word was actually “boasts” as a synonym of the word “claims”. However, we agree it may appear confusing so we have changed the word to “features”

*l. 417: a word is probably missing between "approach" and "in"*

The word “described” has been added to improve clarity.

*l. 458 (Table 3): I was puzzled by the fact that the performances of propionate oxidation pathways via lactate or via hydroxypropionyl CoA cyclical are exactly the same. Can the authors provide a mechanistic explanation about this result?*

This is due to the quantization of the energy recovery and steps. Upon reviewing the pathways, they both have the same number of elementary reaction steps (12 steps), and are capable of recovering the maximum amount of energy possible under the conditions used for the simulation (50 kJ/mol out of 63.3 kJ/mol available). In addition, none of the steps were significant thermodynamic bottlenecks that necessitate the dissipation of additional energy. As such, both pathways achieved the most ideal MDF equal to (63.3-50)/12 or 1.1 kJ/mol.

It is worth nothing that upon changing other parameters, in particular pH_in_ and r_H+/ATP_, the performance of the two pathways diverges from each other.

*l. 486 (Fig. 3): if I understand correctly, Fig. 3 and 7 were obtained by changing the value of epsilon in equation (19). Would it be relevant to indicate the values of epsilon in the Figure?*

Indeed, the Pareto graphs in Fig 3 and 7 were generated by manipulating the value of epsilon as described in equation (19). This has been clarified in the figures’ captions including the range of values of epsilon used.

*In the last part of the manuscript (sections 3 and 4), the authors may want to switch to British English (used in the first part of the manuscript) instead of American English*

The spelling has been corrected to be consistent to British English across the manuscript and revised. In addition, additional grammar revisions have been conducted.

***Reviewer 2:***

*Taha et al. present a methodology for designing and evaluating metabolic pathways, with a special focus on energy yields in the form of ATP and proton translocations. They show that different pathways exhibit different trade-offs between this yield and the thermodynamic efficiency in terms of driving forces, and demonstrate, using a Pareto analysis, how this is especially relevant in cases where energy is scarce such as anaerobic fermentation and the reverse TCA cycle. This work is a useful extension of previous pathway analysis methods based on thermodynamics.*

**In general, it is not completely clear why the authors treat alternative electron carriers so differently than proton translocations. In both cases, not all combinations exist in nature but could potentially be engineered or evolved. Both are discrete choices. Similar to changing the proton stoichiometry, switching from NAD+ to a quinone could boost the driving force at the expense of the amount of energy conserved as ATP. And, finally, alternative carriers can also be dealt with using integer variables - using reaction indicators and adding a constraint forcing that only one can be active at a time.*

We thank the reviewer for the suggested alternative to dealing with electron carrier variants. Our definition of variants and pathways was conceived in terms of biochemistry, i.e. molecules, enzymes or moieties that are present functioning in the pathway. The suggested alternative could also be used as an alternative formulation; however, due to the discontinuity introduced by utilizing indicator variables that force the selection of one constraint, we do not believe there will be a significant improve in computation speed. In addition, the current loop approach allows for analysing individual variants, which allows the identification of all optimal electron carrier combinations.

*Furthermore, in the results regarding the rTCA cycle, it seems that this equivalence was overlooked by the authors. They state that using more energetic electron donors (ferredoxin instead of NAD and NAD instead of quinone) yield better MDFs solutions, which should not come as a surprise. However, cells might prefer using less energetic donors in order to conserve more energy in ATP (or rather spend less of it). Including the potential ATP yield as they did for proton translocations would make the analysis much more valuable.*

The result statements in line 551-555 regarding optimum donors were all equally optimal in terms of energy conserved as ATP. To clarify, of the 54 variants in the pathway, 46 were optimal for energy recovery, and 4-6 of the 46 variants (depending on C_ic_) were also optimal for MDF. All energy expenditure considered in the section were a sum of both the SLP terms and chemiosmotic proton translocation terms.

Regarding the energy of the electron donors, the carrier regeneration reactions against hydrogen are also included in the pathway analysis, meaning that the energy cost to create the more reductive carriers (Ferredoxin, NAD, NADP) is taken into consideration.

** Previous versions of MDF used shadow prices to identify and quantify the reactions which constrain the driving forces in the pathway. It would be helpful to add that information to the analyzed pathways in the results section. These reactions should also be the ones where adding extra driving force via SLP or proton translocation would give the largest benefit. Or, if that is not the case, it could highlight the advantage of this current method.*

The concept of shadow prices as discussed in the original MDF work by Noor et al (2014) seems to be applicable only to continuous linear optimisation problems while ours is mixed integer discrete (now explicitly indicated in the abstract). It is possible to develop an alternative manual algorithm for detection of these bottleneck reactions that works for MILP. Furthermore, Gurobi features an irreducible inconsistent subset algorithm that can aid in this purpose. Reference to the last algorithm is made in lines 612-614 of the manuscript.

** Although the constraints used in the optimization process are written out explicitly and clearly, their justification is often lacking:*

*- Line 278: What is the justification for having a non-zero threshold for* *ΔGr? And why is this necessary given the fact that MDF solutions are anyway far from 0 for all reactions. Also, this constraint does not appear in Table 1 (summary of the LP). Is it converted to "F_i" constants? How are they chosen? If not, where do they come from in the "First Optimization"?*

The non-zero threshold value is precisely the driving force of the reaction. Equation (9) and lines 288-289 detail how that ΔG_min_ eventually becomes the driving force term F_i_. In the first optimisation run, they are set to zero in order to determine the maximum potential energy recovery via proton translocations at no driving force imposed.

*- Lines 317-328: The argument that proton translocation energy cannot exceed SLP is unclear and there is no reference given. Also the claim that leaving it unbounded increases computational time seems reasonable but no quantitative evidence is given and it would be helpful to show that the end results are similar at least for one of the examples.*

The statements have been reworded to avoid any confusion. Leaving the number of protons translocated in a given reaction unbounded makes the solver algorithm search a much bigger integer space, thus increasing computation time.

*- Lines 335-338: Why is it more reasonable to bound the total pool of a conserved moiety? The text states this as an obvious fact without a real explanation. Indeed, the total pool cannot be changed by the pathway itself, but it can definitely be changed by reactions that are not in the pathway and therefore it could make sense to allow it in the optimization step.*

The constraint is founded on the definition of conserved moiety, stated in lines 334-336. Acknowledging that the pool can indeed be changed by reactions outside the pathway, we limit the total concentrations to be between 10^-6^ and 10^-2^ mol/L, rather than having the sum forced to equal a single value. This constraint allows us to avoid a scenario where multiple species from the pool (e.g., acetyl-CoA and propionyl-CoA) are all at the upper bound individually, making the total pool in excess of this bound.

*Minor comments:*

** Line 84: It's not clear to me whether ΔG_diss is the same as the change in Gibbs free energy or minus that. In the equation it seems to be positive, which makes sense, but later in line 88 the driving force is equated to (-ΔG_diss) which implies the opposite.*

This was an overlook by the authors and line 88 was corrected accordingly. The driving force is equal to the ΔG_diss, both of which are equal to the negative of the Gibbs energy change of a reaction.

** Line 137: "terminal electron acceptor for an oxidative pathway is hydrogen". Hydrogen cannot accept any more electrons. Perhaps "electron donor" was meant here?*

We thank the reviewer for spotting this error. We have adjusted the statement to stating “reductive pathway” and “electron donor”.

** Line 194: "in kJ/mol.K" - replace "." with "/"*

The unit display has been corrected to eliminate ambiguity.

*Equation 16: applying an upper bound (but not a lower bound, unfortunately) on a log_sum_exp() function is a convex constraint, perhaps that could be useful here?*

It is possible that the solver algorithm utilizes this fact during its computation. In addition, the lower bound will never come into effect since the individual species are still limited at the lower bound.

** Line 359-361: "no driving force forced energy dissipation was required" -> unclear sentence*

The sentence has been rephrased to better convey the intended meaning.

** Line 441: Shouldn't the MDF value be positive (i.e. +1.5 kJ/mol)?*

This was indeed an overlook. The value of the MDF has been corrected to +1.5 kJ/mol
