# Supplementary material for: Optimal evaluation of energy yield and driving force in microbial metabolic pathway variants
Source: PLoS Comput Biol. 2023 Jul 6;19(7):e1011264. doi: 10.1371/journal.pcbi.1011264 (PMC10353829; doi:10.1371/journal.pcbi.1011264)
Supplement: Attachment — Submitted filename: Response to reviewers R2.docx [file pcbi.1011264.s005.docx]

The authors would like to thank the reviewers and editor for the time and effort in reviewing the revised manuscript and for the additional observations to which we provide our responses below and indications where the minor manuscript edits have been implemented.

***Reviewer 1:***

The authors have addressed my questions and the manuscript is in my opinion well improved, but I think that further clarification is needed for the following points.

First of all, I think that including a discussion about regulation in the conclusion could be of general interest and could make the applicability of the method to other pathways more credible, as enzyme regulation of metabolism is ubiquitous. While it is obvious that an enzyme cannot speed up the rate of a reaction that has no driving force, my question was more about the identification of rate-limiting reaction based on pure thermodynamic considerations (i.e. neglecting enzyme regulation that can also be at the source of ratelimiting reactions). From the reply of the authors, I understand that enzyme regulation is not relevant for the pathways considered and the only rate-limiting effect is thus thermodynamic, but I believe that it should be stated explicitly in the paper – for example, that could be clarified on line 611, where the authors mention that "since the approach is based entirely on thermodynamic principles, the pathway analysis results were obtained with minimum need for assumptions and/or parameter estimations." Then what about pathways where regulation becomes relevant? Since the method is said to be "maintaining a high degree of flexibility to accommodate a large range of additional constraints", I think at least a brief comment could be written about possible extensions accounting for regulation

Although the objective of this study is not a comprehensive dynamic pathway kinetic modelling and it is focused on energy limited pathways that cannot be accelerated beyond what driving forces allow for, we agree it is of value mentioning the role of regulation for kinetic models’ development. We have added a closing paragraph in the conclusions on this topic.

Secondly, the authors say in the response letter that "The value of rH+/ATP used for all results shown in the paper is 10/3", but in the manuscript, this value does not appear and 9/3 is mentioned instead (l. 237)

All simulations were conducted using the 10/3 value indeed. A statement clarifying this was added in line 237 and relevant figure captions.

***Reviewer 2:***

In general, the authors answered the majority of my and the other reviewer's comments. However, a few things could be improved still:

-Regarding the iteration of electron carriers: Indeed, adding extra binary (or integer) variables to an MILP will increase computational time and might (in the worst case) be as slow as looping through the entire combinatorial space (i.e. exhaustively). However, this argument is just as relevant for alternative electron carriers as it is for various proton translocation stoichiometries. If, indeed, no benefit is provided by using reaction indicators, then one can simply give up on integer variables completely and run a series of LPs (iterating the possible proton stoichiometries). This will also have the added benefit of being able to analyze the sensitivity of the MDF to the different reaction energies and metabolite concentration bounds (see my last comment).

From a pure optimisation methodology viewpoint, the suggested approach could be interesting among other possible modifications. However as indicated in the previous comment we aim at keeping a connection with biology and our definition of variants and pathways was conceived in terms of biochemistry, i.e. molecules, enzymes or moieties that are present functioning in the pathway due to them having been expressed by a gene encoding for its presence. In addition, the current loop approach allowed us to analyse individual variants and finding if there are more than one optimal combination. All of this is of relevance in terms of the possible engineering of pathways for applications.

The comment is however relevant and we are indeed currently working on a generalised approach for electron carriers in terms of continuous electron voltage potential to investigate fundamental principles behind the existing electron carriers present in nature. However this is outside the objective of this current study and is a work in progress.

On the other hand, if we were to replace mixed-integer formulations by exhaustively looping through the entire combinatorial space, important disadvantages would be expected since (i) it does not scale up well with number of discrete decision variables, and (ii) a MILP formulation is in general more compact, general and elegant than the exhaustive LP approach so we opted for the mixed approach.

- The upper bound on H+ translocations (based on SLP) is reasonable, but could be explained and discussed more clearly. Relaxing it seems feasible, at least for some examples, and then one could see whether the results change significantly. If not, that is another reason to keep it (saving computation time). If they do, it could lead to an interesting discussion (regarding tradeoffs in evolution). Generally, I agree that if there is enough energy for SLP - that should be preferred by evolution. But it doesn't seem like a strict rule and exceptions to it might exist in nature already.

We thank the reviewer for this insightful discussion. We would like to highlight that we did try both tightening and relaxing this boundary. Relaxing it made no difference to the results, while tightening it to only 2 had a minor impact on the optimum driving force in some cases. Tightening the boundary to a maximum of one proton translocation per reaction significantly lowered both the driving forces and maximum possible energy recovery in most pathways studied. These calculations were not shown for the sake of space and focus as there are one of many possible theoretical considerations to discuss that could make the paper too complex.

- Regarding terms and notations, confusion still remains in several places. For example, in line 289 the authors write "When ∆Gr is negative, that energy is dissipated and is usually referred to as the reaction's thermodynamic driving force." - this phrasing might be confusing (does it suggest that when ∆Gr is positive something different happens?).

Later, ∆Gmin is defined as the minimum required driving force but based on equation (7) it should probably be negative as it is an upper bound on ∆Gr (if it were positive that would be redundant, since ∆Gr < 0 anyway). Is ∆Gmin the optimization parameter that is later denoted B (although B is positive)? If so, the sentence "A threshold minimum required driving force (ΔGmin) *is necessary* for any reaction to proceed" is quite confusing, as it suggests a physical reason rather than the kinetic motivation behind the MDF method.

This observation is correct and needed to be clarified indeed. We added some edits to section 2.6.1., ΔGr is the Gibbs energy exchange (needs to be negative) while the dissipated energy is positive and equals -ΔGr. And the ΔG_min_ is a minimum (in magnitude) of energy change required for a finite sensible rate in a reaction. This has also been corrected in the list of symbols.

Furthermore, isn't B missing from the thermodynamic constraints in Table 1 (otherwise it doesn't seem to have any effect on the MILP)?

B is the lower boundary (smallest value) imposed to all driving forces required in the pathway for any given configuration, which is also a decision variable in the problem formulation (see lines 389-390). As such, it appears in the objectives and boundaries of decision variables section of Table 1 (not constraints). It affects the MILP by being the decision variable being maximised, while satisfying the boundary that all individual driving forces F_j_ are greater than it.

- Finally, regarding identifying bottlenecks, indeed MILP solvers do not provide shadow prices and therefore it is not as straightforward to see which constraints are "active". However, it seems to me that this information should still be possible to get (and the authors suggest a few ways in the discussion). Perhaps after finding the optimal H+ translocation stoichiometry, one could run a standard LP only for (re-)optimizing the concentrations - the optimum should probably be at the same place and the shadow prices would be available then. This is quite a significant omission from the analysis and if it requires little effort to achieve, could be quite informative.

Finding the active constraints in this study becomes relevant to identify the bottleneck reaction step. To achieve that the suggested technique or other approaches such as irreducible inconsistence subsets could be used to obtain this automatically indeed. This post-processing improvement to the method could be considered indeed in future developments. In this study however, bottlenecks were identified directly from the metabolite concentration values proximity to their limits and this was not further pursued.

- The code is indeed freely available on Zenodo. However, there is no documentation or instructions on how to use it. It is ridden with commented out lines that were probably relevant during the study but render it unapproachable to others. "main_nl.m" refers to a non-existing .xlsx file. Please ensure that a fresh download of the repository can be run by other users, clear out the comments, and add a proper README.

A new Zenodo link (with a fresh DOI) has been included, where a short README file has been added. All old commented code lines have been deleted, while comments that explain the purpose of each code section have been kept as they help in clarity.
